# Why Language Processing Recruits Modality Specific Brain Regions: It Is Not About Understanding Words, but About Modelling Situations

**DOI:** 10.5334/joc.124

**Published:** 2020-09-30

**Authors:** Zoé Cayol, Tatjana A. Nazir

**Affiliations:** 1 Univ. Lyon, CNRS, UMR 5304 – Institut des Sciences Cognitives – Marc Jeannerod, Bron, FR; 2 Univ. Lille, CNRS, UMR 9193 – SCALab – Sciences Cognitives et Sciences Affectives, Lille, FR

**Keywords:** Embodied Cognition, Language, Motor Action, Emulators

## Abstract

Whether language comprehension requires the participation of brain structures that evolved for perception and action has been a subject of intense debate. While brain-imaging evidence for the involvement of such modality-specific regions has grown, the fact that lesions to these structures do not necessarily erase word knowledge has invited the conclusion that language-induced activity in these structures might not be essential for word recognition. Why language processing recruits these structures remains unanswered, however. Here, we examine the original findings from a slightly different perspective. We first consider the ‘original’ function of structures in modality-specific brain regions that are recruited by language activity. We propose that these structures help elaborate ‘internal forward models’ in motor control (c.f. emulators). Emulators are brain systems that capture the relationship between an action and its sensory consequences. During language processing emulators could thus allow accessing associative memories. We further postulate the existence of a linguistic system that exploits, in a rule-based manner, emulators and other nonlinguistic brain systems, to gain complementary (and redundant) information during language processing. Emulators are therefore just one of several sources of information. We emphasize that whether a given word-form triggers activity in modality-specific brain regions depends on the linguistic context and not on the word-form as such. The role of modality-specific systems in language processing is thus not to help understanding words but to model the verbally depicted situation by supplying memorized context information. We present a model derived from these assumptions and provide predictions and perspectives for future research.

## 1. Introduction

Since the discovery of mirror neurons, Rizzolatti and Arbib’s influential paper ‘Language within our grasp’ (1998) and Pulvermüller’s paper ‘Words in the brain’s language’ (1999), ideas about embodiment and language processing have entered the cognitive neuroscience literature. The embodied view of language processing holds that brain structures, which traditionally have been seen to serve perceptual, affective, and motor processes, are also recruited for understanding language that refers to perception, emotion, and action. A large number of empirical papers that provide evidence for such **l**anguage-**i**nduced **a**ctivity in **m**odality-specific **b**rain **s**tructures (which we will henceforth abbreviate as LIAMBS) have since been published (for reviews, see e.g., Binder & Desai 2011; Glenberg & Gallese 2012; Fischer & Zwaan 2008; Kiefer & Pulvermüller 2012; Pulvermüller 2018; Pulvermuller & Fadiga 2010; Willems & Cassasanto 2011; Willems & Haggort 2007). However, the role of this activity in behavior remains a matter of dispute. More recent evidence that LIAMBS is not always observed during language processing (for reviews, see e.g., Meteyard et al. 2012; Willems & Cassasanto 2011; see also evidence from brain-damaged patients, e.g., Arévalo, Baldo & Dronkers 2012) has led an increasing number of researchers to conclude that the recruitment of modality-specific brain structures during language processing is ‘optional’ and therefore not essential. In the present position paper, we propose to take a slightly different view on these issues to better assess the potential function of LIAMBS.

### 1.1. The Phenomenon

When Hauk, Johnsrude and Pulvermüller (2004) and Tettamanti et al. (2005) provided the first brain imaging evidence that processing words and sentences that refer to motor actions can trigger (somatotopic) activity in premotor and primary motor structures of the brain, both teams of researchers instantly suggested that their results had something to do with how word meaning is formed in the brain. According to Hauk et al. (2004: 301 in the abstract) *‘These results demonstrate that the referential meaning of action words has a correlate in the activation of motor and premotor cortex’*, and Tettamanti et al. (2005: 278) wrote that *‘… our findings are consistent with the hypothesis that understanding sentences conveying an action-related content requires the contribution of sensorimotor circuits, partially overlapping with those active during the execution and observation of the same actions’*. The rush to such a conclusion is understandable. Words of a spoken language can convey objective or practical meaning. Therefore, a part of the brain activity that emerges during the processing of words and sentences should result from processes that assist in meaning construction. Since premotor and primary motor structures are involved in the planning and execution of motor actions (e.g., Rizzolatti, Luppino & Matelli 1998), the selective recruitment of these structures during action-word processing has thus been interpreted as relating to the elaboration of action-related word content. Since then—that is, right from the start—research on this topic has mainly focused on the *interpretation* that has been given to this intriguing finding and not on the phenomenon itself. The question that dominated the field was ‘Is LIAMBS necessary to understand words?’

Biased by this question, researchers started looking for parallels between motor disorders resulting from brain damage and difficulties in processing action words. While some of these studies showed the expected relation between lesions in motor brain structures and difficulties in processing action-related words (e.g., Albani et al. 2010; Bak et al. 2001; Bak & Hodges 2004; Bak et al. 2006; Bertella et al. 2002; Boulenger et al. 2008; Cotelli et al. 2006; Cotelli et al. 2007; Grossman et al. 2008; Herrera et al. 2012; Riccardi et al. 2020; Silveri & Ciccarelli 2007; Silveri et al. 2012), others did not (e.g., Arevalo et al. 2012; see also Vannuscorps & Caramazza 2019 for results from individuals with Dysmelia).

To date, the available data are not convincing enough to conclude that LIAMBS is fundamental to word recognition, and the topic is becoming somewhat “outdated”. However, the initial observation remains as extraordinary as it was from the beginning: under specific conditions, language processing almost instantly recruits perceptual and motor structures of the brain (e.g., Pulvermüller 2005). Yet, after more than 15 years of research, we still do not know exactly why.

### 1.2. The Question

One obstacle to understanding the function of LIAMBS might be the question that was being asked. Questions matter, however. Suppose we monitor brain activity while the participant perceives a picture of a yellow banana. Among the activated brain structures, we will find a region in the ventral occipital cortex involved in color perception (e.g., Lueck et al. 1989). Is this brain region therefore necessary to recognize the fruit? The answer is ‘no’ because we can recognize a banana in a black and white photograph. Color information might ‘enrich’ the percept of the banana (see Mahon and Caramazza (2008) and Hickok (2009) for such arguments with respect to LIAMBS) and speed up recognition time. However, since we can identify a banana without referring to its color, this information is not necessary to recognize one. Framed within the question of how we recognize bananas, the investigation of the role of color-specific brain regions is thus of little use. However, if we ask instead what color information *serves* when we see a banana, a different picture emerges: the color of the banana allows distinguishing a ripe from a raw fruit. Consequently, although color information is not needed to recognize a banana, it still provides vital information for our actions, as it helps prevent harvesting raw fruits (see Allen 1879; Regan et al., 2001). Note, however, that we can achieve the same goal by referring to information other than color, i.e., by assessing how the fruit smells, how it feels, the particulars of its weight, etc. Color is thus just *one* of several features that can be used to assess the ripeness of the banana.

### 1.3. Therefore, what does LIAMBS serve?

We suggest that like color information for bananas, LIAMBS does not serve to *recognize* or *understand* words. Understanding language is not an end in itself. Rather, in line with pioneering work on perception-action circuits, according to which perception does not serve object recognition per se but instead involves the ‘*gaining of access to motor schemas for controlling interactions with the object*’ (Arbib 1972: 168), we propose that language processing assists in the verification, prediction, and preparation of our interaction with the environment. It is in the service of such functions that language processing recruits modality-specific brain structures.

### 1.4. The structure of our argumentation

The arguments that we will develop in the following sections are based entirely on existing elements in the literature. As a matter of fact, all that we will say in developing our proposal has been said previously in one form or another. We will just restructure these earlier arguments to make it easier to comprehend the entire picture. Note that we will not develop a model of language comprehension. Rather, our proposal concerns *why* and *how* language processing takes advantage of modality-specific brain regions. The position that we defend is that, like color information used for the assessment of the ripeness of bananas, activity in modality-specific brain regions is one of several (partly redundant) sources of information that is used in a (linguistically) regulated manner during language processing. In contrast to Hauk et al. (2004) and Tettamanti et al. (2005), we do not assume that LIAMBS reflects processes related to the elaboration of what a word stands for. Rather, language processing will take advantage of sensory-motor structures to access associative memories via emulators of the motor system, (c.f. Grush, 2004). These associative memories help model the verbally described *situation* (c.f. Situation models; van Dijk & Kintsch 1983; Zwaan & Radvanksy 1998) in order to anticipate and prepare appropriate behavior. To this end, we will take three existing models as theoretical scaffolds that address interactions between language processes and sensory motor information (Section 2). We will then take a closer look at the ‘original’ function of brain structures in modality-specific brain regions that are recruited by language processes (Section 3). Next, we will characterize linguistic conditions that trigger LIAMBS (Section 4). Finally, we will redraw an old picture from a slightly different perspective by suggesting how elements from the three existing models can be combined and extended to better model the function of LIAMBS (Section 5). In the last section (Section 6), we will present future research prospects in this domain.

## 2. Three Models

The three models that will serve our theoretical scaffolds and that we will briefly discuss below include the ‘Action-Perception Circuit’ (APC) model proposed by Pulvermüller (1999, 2013, 2018), the ‘Language and Situated Simulation’ (LASS) theory developed by Barsalou et al. (2008), and a model elaborated by Evans (2009, 2016), with related ideas from Bergen (2012). The first two models have been very instrumental in the domain of embodied language processing. Pulvermüller (1999) was among the first to suggest a neural mechanism for how word-use in the context of objects and actions can lead to associations between neurons in the cortical core language areas and neurons in brain regions involved in motor action and the processing of perceptual information. The LASS theory builds on Barsalou’s (1999) influential Perceptual Symbol System theory, which suggests that the re-enactments of states in modality-specific systems underlie conceptual processing. Finally, Evans and Bergen’s work helps to underline that input from linguistic theories is required for a better understanding of the role of LIAMS during language processing. Note that all models commonly assume that language processing involves the interplay between two representational systems, one that deals with linguistic rules and one that deals with conceptual information. However, the models emphasize different aspects of these components, which allows them to be combined into a single, more elaborated model that we will outline and further develop in Section 5. Our description of the three scaffold models will not be exhaustive, however; it will instead focus on elements that we consider essential for our arguments. Interested readers should refer to the original works by these authors.

### 2.1. The ‘Action-Perception Circuit’ (APC) model by Pulvermüller (1999; 2013; 2018)

Pulvermüller adopts the view that interlinked action-perception representations provide the basis of human cognition and communication (for a recent version of this model see Pulvermüller 2018). At the heart of his theory is the assumption that pre-established neuroanatomical connections between different brain structures allow the development of functional neural networks through Hebbian association-learning, according to the motto: “Neurons that fire together wire together – neurons that are out of sync delink” (Pulvermüller 1999, 2013, 2018; Pulvermüller et al. 2014; see also Galese & Lakoff 2005). Through such ‘Action-Perception Circuit’ (APCs), body actions can be linked to their sensory effects. The vast frontotemporal connectivity via the left arcuate fascicle, for instance, allows the mapping of speech articulatory gestures onto the produced sounds (e.g., Braitenberg & Pulvermüller 1992; Braitenberg & Schüz 1998). Pulvermüller considers this perisylvian circuit as the cortical correlate of spoken word-forms. If, during the acquisition of novel words, a word-form circuit is, for instance, active together with circuits related to the processing of visual object information (e.g., during the acquisition of a concrete noun), Hebbian learning mechanisms will bind the two circuits. Thereafter, the presentation of the word-form by itself will trigger activity in the perisylvian circuit *and* in the visual cortex (see Fargier et al. 2012, 2014 for empirical evidence along this line). Similarly, correlated activity in the word-form circuit and in circuits related to the processing of action-related information or information concerning other modalities will result in the formation of APCs for words that refer to actions, smells, sounds, etc. These ‘embodied’ circuits are essential for establishing links between symbols (words) and the objects/actions they refer to (c.f. the ‘symbol grounding’ problem^1^; Harnard 1990; Searle 1980), and the APC model considers these circuits to be the neural basis of word semantics.^2^

The APC model does not postulate the existence of a special-purpose ‘linguistic system’ (Pulvermüller 2018). Rather, it sees linguistic rule formation as the consequence of correlation-based learning, implemented in a brain with given connectivity structures (e.g., specific short- and long-range connectivity) and prespecified functional properties (e.g., neural units that respond to relationships between events). The way linguistic rules interact with APCs is not further developed.

### 2.2. The ‘Language and Situated Simulation theory’ (LASS) by Barsalou et al. (2008)

The ‘Language and Situated Simulation theory’ (LASS) by Barsalou et al., which shares many features with Paivio’s dual code theory (1971), postulates two independent systems of knowledge representation: i) the ‘linguistic system’ (L), which is responsible for the shallow processing of word-forms, and ii) the ‘situated simulation system’ (SS), which allows the re-enactment of experienced perceptual, motor, and introspective states (Barsalou et al. 2008). The LASS theory assumes that the (L) and (SS) systems both become active when a word is perceived. When the (L) system, whose activity typically grows faster than that of the (SS) system, recognizes a given word-form, a number of associated word-forms that co-occur in natural language become active and provide superficial conceptual information about the target word. In line with Burgess & Lund (1997) and Landauer & Dumais (1997), the LASS theory stipulates that such networks of associated word-forms represent linguistic context. However, it is assumed that the (L) system does not provide profound conceptual information. Rather, conceptual knowledge is specified in the (SS) system through the re-enactment of states in modality-specific systems that are acquired during experiences with the world, the body, and the mind (Barsalou 1999, 2009; Barsalou et al. 2008). Similar to the APC model, the LASS theory assumes that activity in modality-specific brain regions is an essential part of lexical semantics. The model also assumes that hubs in cortical association areas assimilate information across modalities. These hubs, which are equivalent to Damasio’s hierarchically organized convergence-divergence zones (Damasio 1989; Meyer & Damasio 2009), drive the process of re-enactment in the absence of bottom-up stimulation. Barsalou (1999) refers to these hubs or distributed multimodal systems as *simulators* and to the re-enactment of past experiences triggered by these simulators as *simulations*. Since a simulator integrates the content of a category across instances, it also acts as a concept. Like the APC model, the LASS theory places more emphasis on the elaboration of the (SS) system than on the elaboration of the (L) system. However, through its reference to linguistic context theories (Burgess & Lund 1997; Landauer & Dumais 1997), the LASS theory allows predictions about the output of both systems for specific language tasks (e.g., word-associations tasks; see Simmons et al. 2008; Santos et al. 2011).

### 2.3. The model by Evans (2009; 2016) and ideas from Bergen (2012)

In contrast to the first two models, the model suggested by Evans focuses more on the ‘linguistic system’. According to Evans, linguistic communication takes advantage of an evolutionary, prior conceptual system that did not evolve for communication. Similar to the LASS theory, the model postulated by Evans involves two qualitatively distinct representational systems, the ‘linguistic system’ and the ‘conceptual system’, which both contribute to the elaboration of linguistically mediated meaning. Following the proposal by Barsalou (1999), Evans hypothesizes that conceptual representations are contingent on bodily experiences. The ‘conceptual system’ thus holds analog knowledge, i.e., concepts that relate to entities about which we have experiences and retain detailed knowledge. The ‘linguistic system’, by contrast, holds more schematic parametric knowledge that is *unique* to this system. This latter point is crucial and is illustrated in a simple example that we borrow from Evans (2016: 6). Sentences (1) and (2) have the same structure, but in sentence (2), all content words have been replaced by X’s. The function words are printed in bold (i.e., inflections -*ing* and -*s* and lexical items *those, are*, and *my*).

(1) **Those** decorator**s are** ruin**ing my** wall**s**.
(2) **Those** X**s are** X**ing my** X**s**.

Note that despite the missing word-forms, sentence (2) still contains significant semantic content: The first X is an agent(s), the third X is the patient. The first X is performing an action (indicated by the second X) that affects the third X. In line with Bergen (2012; Bergen & Chang 2005), Evans proposes that grammar determines *how* knowledge in the conceptual system is accessed during language processing. Word order and grammatical markers, for example, specify who is doing what action to whom; grammatical aspects indicate whether an event or action just started, is ongoing, or is completed; personal pronouns modulate perspective, etc. According to Evans, the ‘linguistic system’ ‘*has evolved and is designed to exploit those non-linguistic representations for purposes of linguistically mediated communication*’ (Evans 2016: 11). In contrast to the APC model, which considers individual word-forms (by way of their association history) as the driving force for the recruitment of modality-specific brain regions, Evans, and also Bergen (2012; Bergen & Chang, 2005), designate grammar as the decisive element. LIAMBS is not simply triggered by word-forms but follows rules defined by the ‘linguistic system’.^3^

Together, the three models essentially boil down to the following: As stipulated in the LASS theory (Barsalou et al. 2008), linguistically mediated communication involves two different systems. One system, which we will refer to as ‘S_im_’, allows the ‘re-enactment’ or **sim**ulation of bodily experience with the environment, and another system, which we will refer to as ‘L_ing_’, manages **ling**uistic rules. The APC model (Pulvermüller, 1999, 2013, 2018) specifies how, by way of APCs, a given word-form could come to trigger activity in modality-specific brain regions (i.e., via the S_im_ system). The model proposed by Evans specifies how L_ing_ and S_im_ could interact. According to Evans (see also Bergen 2012), the recruitment of S_im_ is governed by the system that manages linguistic rules (i.e., L_ing_). In other words, whether or not an APC that links a word-form to S_im_ will be activated is determined by rules specified in L_ing_. In the next two sections, we will have a closer look at S_im_ and L_ing_ in turn.

## 3. System S_im_: ‘Re-Enactment’ of Bodily Experience

To better understand the role of LIAMBS in language processing, it is helpful to specify exactly what language processes recruit in modality-specific brain structures. Recall that according to the APC model, Hebbian learning mechanisms link a word-form to S_im_, e.g., an action word to brain structures underlying motor action. Because of this link, the presentation of the action word can trigger activity in brain motor structures. Note however that activating an APC for an action word will not prompt the *execution* of that action. When hearing the sentence “Tom signs the contract”, we typically do not produce a hand movement corresponding to the action of signing. The neural activity in brain motor structures that is triggered by an action word is thus not equivalent to the neural activity that drives a real motor action. Therefore, what is it that language processes link to in motor structures of the brain? To answer this question, we will take a quick detour to research on motor control.

### 3.1. Predictive mechanisms in the brain

In his motor simulation theory, Jeannerod (1995, 2001; see O’Shea & Moran 2017 for a recent discussion) suggested that any overt action involves a covert computational stage that includes the goal of the action, the means to reach it, and its consequences. The only difference from a real action is that a covert action is not executed. One critical function of this covert stage is the assessment of the feasibility of the action. Jeannerod’s claim is based, among others, on observations that mentally *imagined* actions retain the same temporal characteristics as the corresponding real actions. Fitts’s law, for instance, which predicts the time required to rapidly move (e.g., with a finger) to a target area as a function of target distance and width, applies equally to real and imagined actions (e.g., Sirigu et al. 1995). Similarly, when asked to estimate the feasibility of an action (e.g., grasping an object placed at different orientations), participants’ response times vary with the orientation of the object in the same manner as the time needed for the execution of the action (e.g., Frak et al. 2001; Fischer & Dahl 2007).

Recent brain imaging studies that used advanced multivariate decoding techniques established that while the overall pattern of neural activity during imagined and executed actions are not the same, the two tasks trigger similar patterns of activity in two regions of the brain: the superior parietal lobule and the dorsal premotor cortex (Zabicki et al. 2017; see also Fillimon et al. 2015; O’Shea & Moran 2017). In motor control, both brain regions (along with the cerebellum) are associated with the elaboration of so-called ‘internal forward models’ or ‘emulators’. An emulator is a mechanism that learns the causal relationship between an action and its sensory consequences through experience (Grush 2004; Wolpert et al. 1995; Zabicki et al. 2017). Emulators can thus predict the outcome of a motor command in terms of the sensory reafference the movement will generate.

Models of action control assume that predicting the consequences of a motor command is necessary because sensory feedback (reafference from sensory receptors in the muscle, skin, and joints, as well as from the visual system) that conveys information about body state is too slow to allow fast corrections of an ongoing movement (e.g., Grush 2004; Pickering & Clark 2014; Wolpert & Flanagan 2001; Wolpert & Ghahramani 2000; Wolpert et al. 1995). Through association learning, the emulator ‘knows’ what the consequence of a given action should be in terms of muscle and joint state. This prediction is used for online monitoring of the ongoing movement in place of the reafferent signals from the perceptual system.

**Figure 1:**
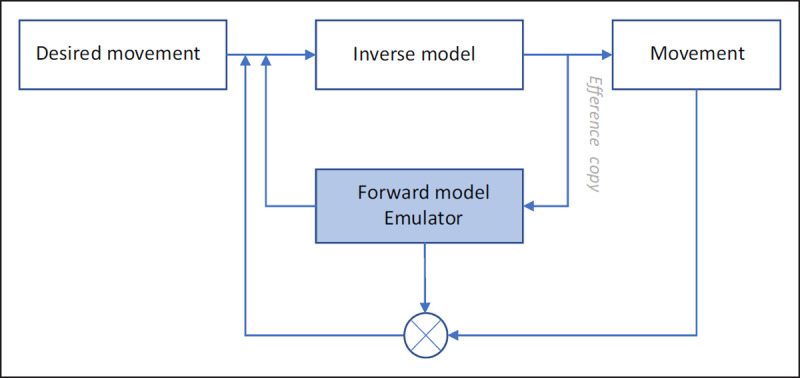
A simple schema of an emulator in motor control. A desired movement is converted into a motor command via the inverse model. A copy of the motor command generated by the inverse model (“efference copy’) feeds into the emulator. Its output is used for internal feedback control. A (delayed) copy of the output of the forward model is compared with the sensory feedback and is used to adapt the emulator and inverse model (modified from Blazquez & Pastor 2013).

Figure 1 represents an emulator schematically. A desired movement (future state or goal) is sent to the ‘inverse model’, which converts this signal into a motor command to achieve that goal. This command is sent to the body to generate movements. A copy of this motor command (the efference copy) is also sent to the ‘forward model’, which predicts the sensory consequences of the motor command. This prediction serves to monitor whether the unfolding action matches the desired outcome. In addition, a copy of the output of the ‘forward model’ is delayed and compared with the actual sensory feedback. This comparison serves to adjust the ‘forward’ and ‘inverse models’ (i.e., correcting the predictions if necessary).

To demonstrate the usefulness of such predictions, stretch out your left arm, palm up. Take a heavy book and place it on your left palm. When your left arm has stabilized, remove the book with your right hand and watch the position of your left hand. You will see that your left hand will hardly move. However, if you ask someone else to take away the book, your left hand will move upwards (e.g. Paulignan et al. 1989; Wolpert & Flanagan 2001; Wolpert & Ghahramani 2000). According to models of action control, the reason for this is that in the first case, the emulators predict the consequence of your self-generated motor commands. This allows compensating for the changing load force due to the removal of the book. In the second case, since someone else takes away the book, your emulators have not received an efference copy of the outgoing motor command. To compensate for the changing load force, you thus rely on visual information only (e.g., observing when the other person is touching the book). Since neural transmission of this visual feedback is too slow, the consequence of the other person’s action cannot be compensated in time. As a result, your left hand reacts to the changing load force and moves upwards. Another role of emulators in motor control is thus to assure coordinated actions despite delays in sensory feedback (Wolpert & Flanagan, 2001; Wolpert & Ghahramani, 2000).

Such predictive mechanisms also have consequences for other modalities (see Clark 2013; Grush 2004). An often-cited demonstration in the visual domain was suggested by Ernst Mach (1896). Mach proposed turning the eyes leftwards and blocking them using firm mastic fixed to the right side of each eye. A command to saccade to the right (which cannot be done properly because of the mastic) would produce the sensation of a rightward shift of the visual scene. This phenomenon suggests that prior to triggering the saccade, emulators predict the state of the visual scene that should be encountered after the execution of the motor command (i.e., the saccade). The mismatch between prediction and the actual state after the saccade (which is caused by physically blocking the eyes) is interpreted by the brain as a movement of the exterior world (see also Helmholtz). Hence, in vision, such predictive mechanisms would allow distinguishing between self-induced and external movements.

A primary function of emulators is thus prediction for the online supervision of actions. However, as suggested by Jeannerod (1995) and elaborated in a very accessible way by Grush (2004), emulators can be ‘run offline’. Decoupled from sensory input and motor output, emulators serve planning and learning by way of *mental imagery*.

### 3.2. Emulators and mental imagery: A proposal by Grush (2004)

By referring to the learning history of a robot designed by Mel (1988), which consists of an articulated arm and a camera that ‘sees’ the arm moving in its environment, Grush (2004) noted how emulators could come to produce ‘mental imagery’. For this to happen, there should be i) a motor system that can interact with the environment (the robot’s arm), ii) a perceptual system (in the present case, a visual system) that monitors this interaction (the camera), and iii) a structure that allows representing and linking the two systems (a connectionist network). In the case of Mel’s robot, this latter structure comprises two bidirectionally interconnected neuron-like units, one that represents the visual information and one that represents the angles of the three joints of the arm. Activity in this motor unit determines the configuration of the arm. By moving the arm through a small representative sample of joint configurations during training, the connectionist network learns the relations between the action of the robot’s arm and the resulting state of its visual field. After this training period, the state of the motor unit allows ‘predicting’ the state of the visual units, and vice versa. As indicated by Grush, the neuron-like units have learned the so-called ‘forward mapping’, i.e., they learned that if the visual state at time t_1_ is v_1_, a motor command m_1_ will result in a visual state v_2_ at time t_2_. Once this mapping is acquired, Mel’s robot can use this forward model to ‘mentally’ determine an optimal trajectory to achieve a visually specified goal prior to executing the movement. For this, the neuron-like units operate offline, i.e., disconnected from the arm and the camera, by using an efference copy of the motor command as input. In other words, the emulator is ‘re-enacting’ or ‘simulating’ a series of potential trajectories prior to executing the optimal action. Note that the forward model computed by the emulator represents the covert stage postulated by Jeannerod (1995), i.e., the stage that accompanies every overt action^4^ (for online monitoring of the action), that can also proceed without executing the action. As pointed out by Grush (2004), such offline use of the emulator is equivalent to ‘mental imagery’.

Another of Mel’s robots described by Grush is also worth mentioning here, as it helps clarify additional terms. This second robot has a visual system and can move around. During training, the robot moves towards or away from, or circles around an object. When the emulator has learned the forward mapping of the motor-visual loop, the robot can engage in offline mental imagery, similar to the first robot. Given its experience with circling around the object, the robot has learned that if the visual state at time t_1_ is v_1_, a motor command m_1_ (e.g., a movement around the object) will result in a visual state v_2_ (e.g., a rotated visual image) at time t_2_. This second robot has thus acquired the ability to perform mental rotation (of the object he circled around). Note that while mental rotation of a visual object is a visual task, the robot had acquired this skill by way of its action.

In discussing this latter robot, Grush outlined how emulators could account for interference effects between two tasks when executed simultaneously. For this, Grush refers to a study by Wexler, Kosslyn and Berthoz (1998), in which participants were requested to mentally rotate a visually present shape while applying a torque to a handle. This dual-task triggered what we could call an ‘action-imagery compatibility effect’, akin to the well-known ‘action-sentence compatibility effect’ or ‘ACE’ in language processing (Glenberg & Kaschak 2002). In fact, the response times of the participants in the mental imagery task were slower when the direction of the rotation that had to be performed with the hand was incompatible with the direction of the rotation that had to be mentally performed on the visual image. By assuming that mental rotation of visual shapes is achieved via the forward mapping performed by (the same) emulators, such interference should indeed be expected.

In summary, the theoretical concept of emulators makes the notions of ‘simulations’ and ‘mental imagery’ more tangible. A simulation occurs when an emulator runs offline to predict the (sensory) consequences of a motor command. Mental imagery results from *conscious* access to these simulations. When two tasks recruit the same emulator to simulate opposing trajectories, each of the tasks will be performed less efficiently. Finally, since emulators serve as the basis for real as well as mentally imagined action/perception, the two tasks recruit partially overlapping representations, i.e., *overlapping brain structures*.

### 3.3. S_im_ and Language processing

Recall that following the models from Barsalou (2008), Pulvermüller (2018), and Evans (2016), we so far postulate two independent systems, S_im_ and L_ing_. S_im_, on the one hand, allows the ‘re-enactment’ or ‘simulation’ of bodily experience with the environment. However, in contrast to the LASS theory, according to which simulations are performed in hierarchically organized convergence-divergence zones, in our proposal S_im_ consists of emulators described in the previous section. L_ing_, on the other hand, manages linguistic rules. In line with arguments developed in the action-based language theory of Glenberg and Gallese (2012), we propose here, that language processing takes advantage of the associative-memory network of S_im_-emulators for simulating (i.e., mentally imagining) a verbally described *situation* (similar ideas have also been developed by Zwaan 2014). Note that mentally depicting the situation described in a sentence such as ‘Tom signs the contract’ will tell far more than provided by the words, e.g., that it probably involves a pen, a sheet of paper, a table, etc. It might even give the reader an idea of the color of the document that Tom signs. Crucially, mentally imaging the action of signing is *not about understanding words*. It is about *modeling situations* in the sense elaborated by Zwaan and colleagues (e.g., Zwaan & Radvanksy 1998; Zwaan, Langston & Graesser 1995; for a recent discussion of these issues, see Zwaan 2016). A situation model (van Dijk & Kintsch 1983; see also Johnson-Laird 1983) is a mental representation of the verbally described state of affairs that includes protagonists, objects, times, places, causality, etc. (Zwaan & Radvanksy, 1998). Such models are needed to coherently integrate verbally communicated content.

On first sight, the distinction that we make about understanding words versus modeling situations might sound trivial. However, the two conditions make different predictions. If LIAMBS were about understanding words, an action word such as “signing” should trigger activity in motor structures of the brain whenever the word is encountered. However, if it is about modeling situations, the same action word may or may not trigger LIAMBS depending on whether the action is *central* to the verbally depicted situation. This latter feature, in turn, is specified in L_ing_.

## 4. System L_ing_: Linguistic Rules and LIAMBS

As mentioned in the introduction, words that refer to perception/action do not always trigger LIAMBS. Hence, solely adhering to a Hebbian association mechanism that embeds lexical items into APCs cannot account for such findings. LIAMBS must therefore be considered in the background of linguistic context conditions that favor its manifestation.

### 4.1. Action-Sentence compatibility effects

The sensitivity of LIAMBS to linguistic contexts has mainly been demonstrated using behavioral studies that assess compatibility effects (e.g., Glenberg & Kaschak 2002; Taylor & Zwaan 2008). In the following we will summarize some of these works that we consider essential for the understanding of the linguistic factors that might govern LIAMBS. In the previously mentioned ACE paradigm (‘Action-sentence compatibility effect’; Glenberg & Kaschak 2002), for instance, participants are exposed to sentences that imply transfer (movement) of a concrete object toward or away from themselves (e.g., ‘You gave the book to Pia’). The participants’ task is to make a judgment about whether the sentence is meaningful by moving the hand toward or away from their bodies to push one of two prespecified response buttons. When the direction of the movement that is implied in the sentence and the direction of the movement for the response are compatible, response times are shorter than when they are incompatible.^5^ The ACE is interpreted as showing that understanding the action word in the sentence recruits brain mechanisms that are involved in the execution of the depicted action. If language processing takes advantage of S_im_-emulators, this is indeed what we should expect. However, as Glenberg and Kaschak (2002) and Bergen (2012; chapter 5) pertinently pointed out, since it matters whether *you* gave the book to Pia (this speeds up a movement away from you), or *Pia* gave the book to you (this speeds up a movement towards you), grammatical cues such as subject/object obviously modify the way LIAMBS is triggered: reversing these cues drives the effect of the action in the opposite direction.

A number of linguistic conditions have been tested using such or similar paradigms. Zwaan and colleagues (e.g., Zwaan & Taylor 2006); Taylor & Zwaan 2008; Zwaan, Taylor & De Boer 2010), for instance, used a knob that could be turned clockwise or counterclockwise to reveal successive portions of a sentence that implies a clockwise or counterclockwise manual rotation (e.g., ‘He turned the key to start the car’). Again, interference effects were observed when the direction of the manual rotation was incompatible with the direction of a verbally depicted movement of the hand. Data from this group were among the first to demonstrate that LIAMBS cannot be fully accounted for by the associative-leaning mechanism proposed in the APC model. Rather, following their ‘Linguistic-Focus Hypothesis’, Taylor and Zwaan (2008) suggested that the involvement of modality-specific brain regions during language processing hinges on the *focus* of the linguistic message. Hence, in a sentence such as in (3) described below, evidence for motor activity is seen while participants process the action word ‘opened’ but also during the processing of the ensuing action-modifying adverb ‘slowly’; this is because the adverb maintains focus on the action. The latter phenomenon disappears when the action-modifying adverb is replaced by an agent-modifying adverb (e.g., carefully) because the latter adverb focuses on the protagonist’s state of mind.

(3) He selected unleaded at the gas station. He placed his hand on the gas cap, which he **opened** slowly.
(4) John **closes** a juice bottle, and Jim **[ ]** a lemonade bottle.

Relatedly, using the same paradigm, Claus (2015) investigated how verb gapping affects the motor system. Verb gapping is the omission of repeated instances of a verb from conjoined sentences, as indicated by the ‘[ ]’ in sentence (4). For these types of sentences, Claus reported a compatibility effect between the linguistically conveyed action and the manual rotation of the knob for both the overt verb (e.g., closes/opens a juice bottle) and the gapped verb. Again, this kind of finding cannot be accounted for by APCs in the way proposed by Pulvermüller (1999, 2013, 2018).

### 4.2. The grip force sensor

Another series of experiments tested the impact of linguistic context on LIAMBS using a grip force sensor (held between the thumb and index finger; Frak et al. 2010; Aravena et al. 2012, 2014; Nazir et al. 2017; Reinecke, et al., submitted). This method allows the monitoring of grip force variations while participants listen to spoken sentences. Indeed, subtle but selective grip force modulations (not under voluntary control) are seen during the processing of language. When a sentence refers to a manual action—but not otherwise—a significant enhancement in the grip force level is observed starting within 200-500 ms after the onset of the action word. Such an involuntary increase in grip force results from the overflow of language-induced cortical motor activity to the muscles (Frak et al. 2010; Nazir et al., 2017). Hence, similar to event-related potentials measured by means of electroencephalography, the grip force paradigm allows the online monitoring of brain activity as it unfolds in the primary motor cortex (M1).

Using this paradigm, Aravena et al. (2014) demonstrated that even a novel word-form that has never been linked to circuits related to the processing of action information can activate motor brain structures when the linguistic context suggests a manual action. Hence, in a sentence such as (5), a word-form that had never been encountered before (i.e., ‘to capame’) will provoke activity in M1, similar to the action word ‘to sign’. By contrast, when embedded in a volitional sentence form such as in sentence (6) or in the context of negation such as in sentence (7), the action word ‘to sign’ will cease to trigger activity in M1 (Aravena et al. 2012, 2014; see also Zwaan, Taylor & Boer 2010).

(5) With his black pen, Tom **capame** the contract.
(6) Tom wants to **sign** the contract.
(7) Tom does not **sign** the contract.

What these series of experiments suggest is that language processing takes advantage of modality-specific brain regions in a ‘rule-driven’ manner. LIAMBS does not seem to reflect processes related to the elaboration of the meaning of individual words because modality-specific brain structures can remain silent despite the presence of words that refer to actions or perception. Rather, what seems to matter is whether or not the action is actually present in the *situation* described in the sentence. While in sentence (5) it seems obvious that Tom is performing an action with the hand, sentences (6) and (7) do not give any indication about what Tom is actually doing. The situation described in these latter sentences does not contain a bodily action, and thus the motor cortex does not respond. Note that despite the absence of measurable activity in the motor cortex, in reading sentence (6), we nevertheless know what Tom wants to do, and in sentence (7), we know what he is not doing. In other words, the meaning of the action word is elaborated without the (measurable) contribution of brain motor structures.

In a series of experiments that used the same paradigm, Reinecke et al. (submitted) recently compared the use of information embedded in a presuppositional factive verb construction (sentence (8)) to that of a non-factive verb construction (sentence (9)). The factive verb “knows” in sentence (8) presupposes the truth of its complement clause, i.e., that Peter irons his shirt, and asserts that Louise is certain that Peter is ironing his shirt (Egre 2008). Presupposed information is considered true information to which the speaker is committed (Peters 2016). By contrast, a non-factive verb construction such as in sentence (9) imposes no constraint on the truth-value of the embedded that-clause.

(8) Louis knows that Peter **irons** his shirt.
(9) Louis believes that Peter **irons** his shirt.
(10) It is Peter who **irons** his shirt.

The result of this study showed that the action verb (‘to iron’) triggers an increase in grip force in the factive (i.e., when Louise *knows*) but not in the non-factive (i.e., when Louise *believes*) condition. Hence, even the truth-conditional status of an embedded clause modifies LIAMBS. Finally, action verbs in it-cleft sentences such as in (10) do not trigger LIAMBS either, because the relevant information in this sentence is not the action, but Peter (i.e., it asserts that it is *Peter* and not Mary or Paul).

All these findings thus suggest that language processing takes advantage of modality-specific brain regions only in conditions in which the action/perception is the primary ‘linguistic focus’ (in the sense used by Taylor & Zwaan 2008) of the verbally depicted *situation*. Whether or not this is the case is specified by linguistic rules, i.e., grammar. Or, to cite Bergen (2012: 118): ‘*Grammar appears to modulate what part of an evoked simulation someone is invited to focus on, the grain of detail with which the simulation is performed, or what perspective to perform that simulation from*.” And grammar belongs to L_ing_.

## 5. Why Language Processing Recruits Modality-Specific Brain Regions

We will now redraw an old picture of LIAMBS from a slightly different perspective by combining elements from our scaffold-models and the concept of emulators.

Recall that our scaffold-models commonly assume that language processing involves the interplay between two representational systems, one that deals with linguistic rules and another that deals with conceptual knowledge. The latter is specified through the re-enactment of bodily experience in modality-specific systems. Instead of this bipartition, we (I) assign a pivotal role to the linguistic system, L_ing_, and (II) regard the brain’s modal system(s), S_im_, as just *one* among several nonlinguistic brain systems that are exploited by L_ing_. L_ing_ is pivotal because it holds instructions (grammar/rules) on how to use these nonlinguistic brain systems for the purpose of language processing. S_im_ is just one among other brain systems exploited by L_ing_ because we can understand sentences such as ‘Tom wants to sign the contract’ without engaging motor brain structures (c.f. Section 4.2). Linguistic meaning construction must therefore (also) occur elsewhere. In addition to S_im_, we propose that L_ing_ will also take advantage of brain systems of statistical learning, i.e., systems that allow the extraction of distributional properties from sensory input (for recent reviews see Conway 2020; Frost et al. 2015). These latter systems, which are functionally equivalent to the (L) system in the LASS theory, exploit information related to word-use across spoken and written language, and provide complementary as well as redundant information to S_im_ (see later). Another potential brain system that might also be exploited by L_ing_ is the theory-of-mind network (e.g. see van Ackeren et al. 2012; Bašnáková et al. 2014; Hagoort & Indefrey 2014), which we mention here without further elaboration, to emphasize that L_ing_ is likely to parasitize a number of different brain mechanisms. Finally, on the grounds that brain activity (in motor structures) triggered by the processing of a verbally described action does not prompt the execution of the action, we propose (III) that S_im_ consists of motor system emulators.

The L_ing_-centered configuration of our proposal is designed to draw attention to the role of grammar in understanding LIAMBS and to relax S_im_ from its vital role in language processing. As we will show, this modified configuration leads to a different conception of the function of LIAMBS.

### 5.1. The L_ing_-S_im_ model: What LIAMBS serve

**Figure 2:**
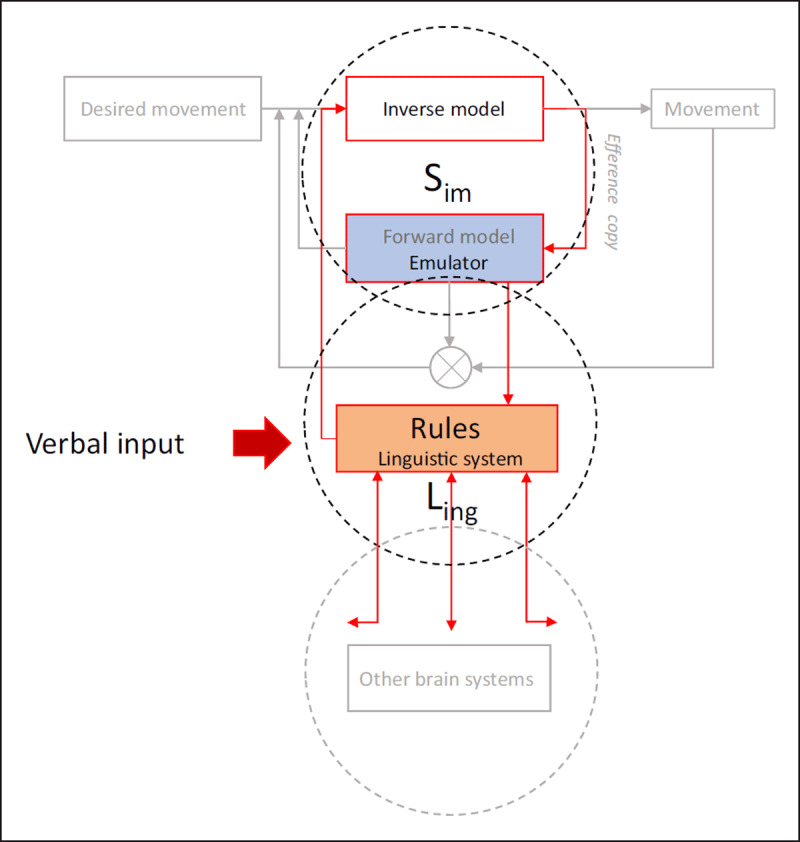
A schematic presentation of the L_ing_-S_im_ model displayed with the schema from Figure 1. The central element of the model is the linguistic system L_ing_, which coordinates the emulator S_im_. S_im_ is decoupled from the rest of the motor loop. Language processing always starts in L_ing_, which holds schematic parametric knowledge (rules/grammar) and provides linguistic cues for how to understand a verbal message. When L_ing_ detects that the verbally depicted situation focuses on an action or a perceptible entity, it will initiate simulations in S_im_ by way of APCs. The link between L_ing_ and S_im_ is bidirectional, allowing the conversion of associative memories into a format that is suitable for language use (see text). Note that L_ing_ exploits nonlinguistic brain systems other than S_im_. The arrows pointing away from L_ing_ at the bottom of the figure serve to suggest such links and highlight that we model only one particular aspect of language processing that specifically addresses the function of LIAMBS.

In Figure 2, we outline S_im_ from the scheme displayed in Figure 1. We further add L_ing_ to the figure and suggest how language processing may interact with the modality-specific brain structures represented by S_im_. Recall from Section 3.2 that we use the term ‘simulation’ to refer to a condition in which an emulator runs offline, and the term ‘mental imagery’ to refer to the conscious access to these simulations. We assume that when S_im_ is ‘exapted’ (c.f. Gould & Vrba 1982) for the purpose of language processing, S_im_-emulators run in the ‘mental-imagery-modus’. With the exception of the subtle overflow of cortical activity to the muscles, which we observe with the grip force sensor (Section 4.2), the processing of action-related language will thus not cause the execution of the action.

Note, it is fallacious to assume that if the brain is ‘running simulations’, a mechanism that ‘reads’ or ‘interprets’ those simulations is required. Recall the heavy-book example in Section 3.1. According to models of motor control (e.g., Wolpert & Flanagan 2001), our left arm compensates for the action of our right hand because predictive information provided by emulators is fed back into the motor loop. In this model, there is no ‘interpreter’. Simulations provided by emulators are *used* in relation to a specific goal. Accordingly, if L_ing_ takes advantage of S_im_-emulators, the simulated information is used to better apprehend the verbally depicted situation.

As indicated in Figure 2, language processing always starts in L_ing_. When L_ing_ detects that the verbally described situation focuses on an action (or a perceptible entity^6^), it initiates simulations in S_im_ by way of APCs (c.f. Pulvermüller 2018). Whether the interaction between L_ing_ and S_im_ happens through excitatory or (the release of) inhibitory signaling is an empirical question (e.g. see Beltran et al. 2019; De Vega et al. 2016). However, when the linguistic focus is not the action, language processing will proceed *without* the contribution of modality-specific brain structures. This is in spite of the presence of words that refer to motor actions. For example, when L_ing_ detects markers for volition or negative polarity, as in sentences (6) and (7), it will not engage S_im_ during the processing of the ensuing action verb.

We further assume that the link between L_ing_ and S_im_ is bidirectional. As stated earlier, mental imagery (i.e., the conscious access to simulations of emulators) allows the recollection of (experienced) information. The well-known example in which a person is asked to state the number of windows in his or her house illustrates this aptly: while the individual can rather quickly name the capital of France or the month when Christmas is celebrated, he or she might need quite some more time to determine the number of windows in his or her own house. However, after *mentally* walking through the house, the individual can come up with the correct answer. This illustrates two things. First, the information about the number of windows is stored in the person’s brain (otherwise it could not be retrieved). Second, by running through such a mental simulation, this information can be transformed into a format that is suitable for language use. The link from S_im_ to L_ing_ enables this transformation.

In the L_ing_-S_im_ model, L_ing_ is thus the central element that holds schematic parametric knowledge (rules/grammar) unique to this system and that provides linguistic cues for how to understand a verbal message (see Section 2.3). Using these rules, L_ing_ coordinates language processing by exploiting brain systems that evolved for purposes other than communication, e.g., emulators of the motor system (S_im_). As stated earlier, we assume that S_im_ is just one of several nonlinguistic brain systems exploited by L_ing_. In line with the LASS theory, we suppose that L_ing_ will also take advantage of brain mechanisms of statistical learning to recover information from the verbal input. In fact, computational linguistic analyses have demonstrated that the statistical distribution of words across spoken and written language carries an impressive amount of information. Hence, word co-occurrences capture categorization information (Louwerse 2011), predict geographical information (Louwerse & Zwaan 2009), and rank concepts of time in a temporally appropriate order (Louwerse et al. 2006). Word co-occurrences also predict iconic relations (Louwerse 2008), motor affordances (Louwerse 2007), and deixis (Louwerse & Van Peer 2009). With knowledge of a set of initial words (which would satisfy the ‘symbol grounding’ requirement), important aspects of meaning can thus be extracted computationally from the linguistic stimulus (Louwerse 2011; see also Andrews, Vigliocco & Vinson 2009; Johns & Jones 2012). S_im_ is thus to language processing as color information is to the assessment of the ripeness of bananas, i.e., *one* among several options. Associative memories provided by statistical learning mechanisms are another option. The arrows pointing away from L_ing_ at the bottom of Figure 2 serve to suggest such links to other brain systems, and highlight that we model only one particular aspect of language processing that specifically addresses the function of LIAMBS.

### 5.2. What does LIAMBS serve?

If L_ing_ can make use of various brain mechanisms during language processing (e.g., S_im_-emulators, brain mechanisms of statistical learning), and if access to these mechanisms is governed by grammar/rules, it is likely that the information obtained through these different systems contributes differently to the elaboration of the verbally conveyed content. Along this line, Connell (2019; see also Andrews et al. 2009; Barsalou et al. 2008; Louwerse 2011) pointed out that linguistic distributional data do not necessarily represent statistical patterns in how entities and events occur in real-world experience. Our sensory and motor experience related to the term ‘democracy’, for instance, does not easily capture the relationship between ‘democracy’, ‘freedom’ and ‘human rights’. Such relationships emerge through language use. Language-based knowledge can thus provide a qualitatively different form of information from that which emerges through sensory and motor experiences (c.f. Connell 2019). In parallel, experience-based knowledge (via emulators) can provide information that has not (yet) been encoded in verbal form (recall the example with the mental counting of windows in your house). Hence, it is typically easier to just state that, e.g., ‘The new climbing carabiner is shaped like the body of a guitar’ (which could then trigger the mental image of a guitar) rather than to verbally describe the form of the carabiner. However, while this difference is generally acknowledged, many theoreticians have argued that language-based knowledge is ‘shallow’, implying that without sensory and motor simulations, the elaboration of the verbal content is incomplete (e.g. Barsalou et al. 2008; Connell 2019; Louwerse 2011). In contrast to this view, we suggest that by exploiting various brain mechanisms in a rule-based manner, L_ing_ coordinates language processing in a way that is *optimal* for the task at hand.

Recall that as demonstrated in the section about action words that are embedded in negative or volitional linguistic contexts (Section 4.2), we do understand sentences that contains action words without the (measurable) involvement of cortical motor structures (c.f. sentences (6) and (7), which do not provoke LIAMBS). Assuming that the lexical meaning of the action word ‘to sign’ is the same in sentences such as ‘Tom signs the contract’ and ‘Tom wants to sign the contract’, selectively engaging S_im_ during the processing of one (affirmative) but not the other (volitional) sentence probably has a purpose. As illustrated with the help of Mel’s robots (Section 3.2), emulators allow the retrieval of a large amount of knowledge associated with a motor action. In the affirmative sentence context (which, as specified by L_ing_, focuses on the action), S_im_-emulators can therefore help obtain action-related information that is not present in the verbal stimulus, e.g., that Tom is probably sitting at a table and that the color of the document that Tom signs is probably white. This ‘filling in’ of nonexistent information serves to model the described situation for adapted behavior. In contrast, retrieving the same information in the volitional sentence context (which does not focus on the action) would distract from the main message, i.e., that Tom *wants* something. In this context, engaging S_im_ for the action word ‘to sign’ (i.e., modeling that Tom is probably sitting at a table and that the document is probably white) could distort the interpretation of the communicative act. However, owing to L_ing_ and its complementary options (e.g. language-based knowledge), we still understand the sentence. Therefore, LIAMBS does not serve to understand *words* (because S_im_ can remain silent despite the presence of the action word), but it helps to model the verbally depicted *situation* (because S_im_ provide context information when L_ing_ specifies that the depicted situation is about the action).

### 5.3. Consequences and predictions

From this perspective, LIAMBS thus loses its vital status for language processing as defined by influential theories of embodied language processing, i.e., the LASS theory (Barsalou et al. 2008) and ACP model (Pulvermüller 1999, 2013, 2018). At the same time, this modified picture opens up new research avenues as it allows more refined predictions about the manifestation of LIAMBS during language processing. First, if LIAMBS is under the control of L_ing_, it should not be systematically observed whenever words that refer to action or to perceptual entities are processed (c.f. ‘flexibility’; Meteyard et al. 2012; Willems & Cassasanto 2011). By emphasizing the role of grammar, the L_ing_-S_im_ model allows the specification of conditions that trigger LIAMBS on the basis of linguistic factors. A general rule could be that LIAMBS will only manifest when the action or perceptible entity is presented as actually true or ‘veridical’ in the verbally described situation (c.f. the linguistic notion of ‘veridicality’; e.g., Giannakidou 1998). An expression is ‘veridical’ with respect to some proposition whenever it entails the truth of the proposition. In general, interrogative, negative, volitional, and imperative constructions are not veridical and should therefore not provoke LIAMBS. However, further research is needed for a better insight into the rules governed by L_ing_ because linguistic focus, in the sense of Taylor and Zwaan (2008), is another factor that modifies LIAMBS. Second, motor disorders resulting from brain damage do not have to correlate with difficulties in processing action words because action-word knowledge can also be inferred from the distributional properties of words across written and spoken language (e.g. Louwerse 2011; Andrews, Vigliocco & Vinson 2009; Johns & Jones, 2012). However, depending on what brain mechanism is affected by the lesion, more refined testing could reveal that language processing in this population is nonetheless qualitatively different than that in a healthy population. We will return to this point in Section 6. Third, by specifying that simulations are performed by emulators of the motor system, the L_ing_-S_im_ model establishes direct links between motor coordination and language skills. Such links have been discussed in the developmental literature, in particular with respect to certain developmental language disorders (for a review see, e.g., Hill 2001; Sanjeevan et al. 2015). We will also return to this point in Section 6. Finally, the L_ing_-centered structure of our proposal also invites reflections on the nature and function of other potential brain systems that might be exploited for language processing, and on how L_ing_ orchestrates the use of these different systems.

### 5.4. Relation to other theories that model LIAMBS

As we stated in the introduction, all of the above mentioned has been said before in one form or another. Our proposal is largely based on ideas developed by Barsalou and colleagues, particularly in the LASS theory (Barsalou et al. 2008). We replaced Barsalou’s ‘simulators’ or (SS) system with ‘S_im_-emulators’ as described by Grush (2004), which could turn out to be just another way of implementing the same idea (see Glenberg & Gallese 2012). Recall, however, that Barsalou’s ‘simulators’ integrate the multimodal content of a category across instances and function as concepts (Barsalou, et al. 2003). Whether the situational information provided by ‘S_im_-emulators’ should be considered as part of (e.g., Glenberg & Gallese, 2012) rather than a means of accessing conceptual knowledge requires future reflection. Since the word ‘to sign’ does not engage motor system emulators in the same way when used in affirmative and volitional constructions (see Section 4.2), we need to clearly define what we mean by conceptual knowledge. Note also that the mental imagery content that we can simulate when hearing the sentence ‘Tom signs the contract’ can be replaced by the real situation of observing Tom signing the contract. Is that visual scene part of our conceptual knowledge of signing? Along this line, Barsalou, et al. (2003: 84, Box 1) had noted that ‘*The study of conceptual processing will be best served by discovering and describing the relevant mechanisms, rather than arguing about the meaning of lay terms such as concept.’* The L_ing_-S_im_ model is part of an effort to describe such relevant mechanisms.

We also incorporate ideas developed by Evans (2016) and Bergen (2012), by emphasizing that the ‘linguistic system’ L_ing_ provides information that is unique to this system (see Section 2.3). Moreover, as suggested by Evans, we gave a pivotal role to L_ing_ in controlling other brain mechanisms for the purpose of linguistically mediated communication. By contrast, we relegate the function that is ascribed to the linguistic system (L) in the LASS theory (i.e., recovering knowledge from the statistical distribution of words across spoken and written language) to nonlinguistic brain mechanisms underlying statistical learning. Like S_im_, this latter system is also controlled by L_ing_. The elaboration of how L_ing_ coordinates such a system and how this system is implemented in the brain is a next step towards the conception of a full-fledged theory of language processing.

Finally, the idea that motor system ‘emulators’ are involved in language processing has also been previously proposed by several authors (e.g., Clark 2013; Glenberg & Gallese 2012; Grush 2004; Pickering & Garrod 2013; Pickering & Clark 2014). In their ‘Integrated Theory of Language Production and Comprehension’, Pickering and Garrod (2013), for instance, suggested that ‘emulators’ could provide a mechanism for coordinating dialogs through alignment in speaking. The action-based language theory from Glenberg and Gallese (2012), which does not postulate an independent linguistic system, is built entirely on emulators to account for substantial aspects of human language, from acquisition to comprehension and production. In their work on this theory, Glenberg and Gallese (2012: 908) explicitly noted that ‘*In the terminology of classic cognitive science, the predictor* [forward model] *corresponds to a mental model (Johnson-Laird 1989), and in the terminology of embodied cognition, the predictor* [forward model] *corresponds to a simulator (Barsalou 1999).’* According to these authors, language comprehension is *‘the process of fitting together actions suggested by the linguistic symbols’* (Glenberg & Gallese 2012: 916). In other words, language comprehension *is* the output of the emulators. Note, however, that the data in the literature are not compatible with the notion that we always need the output of motor emulators to understand language (see Sections 1.1 and 4.2). Recall that language processing takes advantage of modality-specific brain regions only in conditions in which the action/perception is ‘veridical’ and/or the ‘linguistic focus’ of the verbally depicted situation. This feature suggests that simulations are used to provide and emphasize information that is not available in the verbal stimulus (see also Zwaan 2014) to help optimize interactions with our environment.

## 6. Perspectives for Future Research

In this last section, we will describe novel research perspectives related to our proposal.

### 6.1. When S_im_ is disconnected from L_ing_

Recall that S_im_-emulators do not serve the elaboration of what a word stands for but provide access to associative memories that help apprehend a verbally depicted situation. Recall also that L_ing_ has other options than S_im_ for processing language. Neutralizing S_im_ should therefore not erase word-knowledge per se but instead affect language processing in a different way.

According to the L_ing_-S_im_ model, mental imagery results from the conscious access to simulations in S_im_ (c.f. Grush 2004). Individuals who lose the ability to produce mental imagery through brain damage (e.g., Moro et al. 2008; Zeman et al. 2010) or individuals who never acquired this ability (c.f. congenital aphantasia; Zeman et al. 2015), could therefore provide a promising testing ground for our model. In a study by Moro et al. (2008), for instance, a patient who had lost the ability to produce mental imagery anecdotally reported that she had difficulty ordering lunch or appetizers (e.g., when asked ‘Would you like crisps or peanuts?’) because she could no longer visualize them. However, the patient had no problems when actually presented with the food-items, testifying to her intact semantic knowledge. According to our model, when deprived of S_im_, L_ing_ can still retrieve knowledge about words, e.g., from the statistical distribution of words across spoken and written language (c.f. language-based knowledge). However, in the particular case of crisps and peanuts, a number of common words are associated with both appetizers (e.g. salty, crispy, roasted/fried, slightly oily, yellow-brown color, etc.). If the respective web of associated words for crisps on the one hand and for peanuts on the other hand are not sufficiently elaborated (which depends on the individuals’ word-acquisition history), language-based knowledge alone might not allow to distinguish between the two food-items. Here, mental imagery (i.e., S_im_) will provide the distinctive elements that allow one to decide between the alternatives. Deprived of S_im_’s associative memory, the patient will therefore have difficulty solving the task. Hence, if the phenomenon reported anecdotally by Moro et al.’s patient can be confirmed, mental imagery deficits can affect normal language use. This is not because language-based knowledge is ‘shallow’ but rather because the web of associated words is not sufficiently developed.

The complementary picture to patients who lose mental imagery after brain injury is congenital aphantasia. Congenital aphantasia is characterized by the inability to voluntarily use mental imagery (Zeman et al. 2015). Individuals with congenital aphantasia are not identified as a population with language deficits. It is likely, however, that this population processes language in a different way than individuals who can use mental imagery do. If congenital aphantasia results, for instance, from poor emulators,^7^ or from the absence of wiring between L_ing_ and S_im_, this population may have developed alternative strategies and rely heavily on language-based knowledge for processing language. If this assumption is correct, it is likely that they have higher verbal skills than the average population. Individuals with congenital aphantasia thus present a promising population for characterizing the language-based knowledge system and its underlying neural basis.

In short, we believe that in a typically developed brain, language processing takes advantage of S_im_-emulators at the expense of fully developing language-based knowledge. This happens because brain connectivity allows it to do so and because exploiting S_im_ is very convenient. Consequently, when deprived of S_im_ after language is acquired (e.g., following a brain injury in adulthood), a deficit in language use might be observed. By contrast, no deficits will be seen if L_ing_ has never had the opportunity to exploit S_im_ (e.g., congenital aphantasia), because L_ing_ will use alternative strategies from the start.

### 6.2. Poor S_im_-emulators

Another line of research that is motivated by the L_ing_-S_im_ model relates to the documented comorbidity between unexplained language problems (c.f. Bishop 2014) such as ‘specific language impairment’ or SLI (i.e., delayed or disordered language development for no apparent reason), and ‘developmental coordination disorder’ or DCD (i.e. a motor skill disorder resulting in difficulty in mastering simple motor activities such as tying shoelaces or going down stairs) (for an overview, see Hill 2001; Sanjeevan et al. 2015).

Children with DCD have reduced mental motor imagery skills (e.g., Barhoun et al. 2019) and show poor online control during action execution (e.g., Hyde & Wilson 2011). Given that mental imagery skills provide insight into our ability to engage motor system emulators, it is assumed that children with DCD have a deficit in ‘internal forward modeling’ (Adams et al. 2014; Barhoun et al. 2019). In other words, they have poorly developed S_im_-emulators. If S_im_ is exapted for language processing, such dysfunction should transpire during language processing. The L_ing_-S_im_ model thus predicts a link between motor and language skills. Moreover, it allows us to specify that this link should be particularly evident for verbal content that relates to sensory and motor experiences (i.e., highly imageable verbal content) because this is the content provided by S_im_. In a first attempt to test this hypothesis, we recently determined mental motor imagery and word-definition skills in a group of 30 normally developing children. Our results confirmed that mental motor imagery predicted word-definition performance for high imageable words but not for low ones (Cayol et al. in press).

### 6.3. S_im_-emulators, mental training, and consumption of literary fiction

Finally, the L_ing_-S_im_ model also allows us to address an issue that, on first sight, appears far-fetched: The potential benefit of consuming narrative texts for a reader.

Research in the motor domain has established that the offline use of S_im_-emulators can have measurable effects on human performance. Explicit mental-motor-imagery, for instance, is used by athletes to improve their motor skills (e.g., Mizuguchi et al. 2012). Similarly, musicians use kinesthetic mental imagery as a complement to actively playing an instrument (Lotze 2013). Mental motor practice is also frequently used for motor rehabilitation purposes in a variety of neurological disorders (e.g. Jackson et al. 2001). Similarly, visual-mental-imagery contributes to high-level cognitive functions, including navigation and spatial planning (Pearson et al. 2008). As in the motor domain, the content of visual imagery can selectively influence perception (e.g., Craver-Lemley & Reeves 1992; Ishai & Sagi 1997; Pearson et al. 2008). If language processing exploits S_im_-emulators and triggers mental imagery, consuming narrative texts might therefore have similar enhancing effects for the reader, provided that the texts use linguistic/stylistic elements that strongly engage S_im_.

Following Mar and Oatley (2008; see also Oatley 2016), one of the major objectives of narrative texts is the simulation of the self in the social world. This happens through the process of identification and involves perceiving the story from the perspective of the narrative subjects i.e., by adopting his/her goals, beliefs, and values. All these elements are part of the situation model that we develop while reading a narrative text. According to Van Krieken et al. (2017), stylistic/linguistic elements that modulate the process of identification include the use of: (I) personal pronouns (1^st^ and 3^rd^ person perspectives) and whether the narrator is part of the narrative events; (II) verb tense and deictic elements (a readers’ spatiotemporal identification is facilitated by the use of past perfect and present tense because these tenses reduce the temporal distance between character and reader); and (III) verbs that express the viewpoints and actions of narrative characters because these verbs allow using the perceptual, emotional, and cognitive perspectives of the character to mentally represent what she/he perceives, thinks, feels, believes, etc. Recent studies have indeed shown that subtle differences in the form of a verbal utterance can have differential effects on activity in modality-specific brain regions. Yao, Belin, and Scheepers (2011), for instance, showed that reading direct speech (e.g., ‘Luke said: ‘God, that movie was terrible! I’ve never been so bored in my life.’), activates voice areas in the auditory cortex to a higher degree than reading indirect speech (e.g., ‘Luke said that the movie was terrible and that he had never been so bored in his life.’). Readers are thus more likely to engage in mental simulations of the reported speaker’s voice when reading direct speech then when reading meaning-equivalent indirect speech statements. The use of particular linguistic/stylistic elements in writing might thus modulate the extent to which we engage S_im_, which could explain why the work of some writers has longer-lasting effects on the reader than that of others.

## Footnotes

The ‘symbol grounding’ problem refers to the question of how symbols (e.g., words) get their meanings; i.e. how symbols connect to the things they refer to.

In addition to such modality-specific circuits, Pulvermüller (2018) specifies that multimodal convergence zones that serve as hubs between distant brain regions can give rise to more abstract semantic representations.

The model proposed by Evans (2016) is far more complex than described here. However, for the present purpose, the elements mentioned here provide sufficient information for our arguments.

In the case of Mel’s robot, the output of the emulator was not fed back into the motor loop to supervise and guide the motor output.

Note, however, that a recent pre-registered multi-lab study failed to replicate the ACE (https://osf.io/ka2ph/).

Recall from the examples with Mel’s robots that emulators reciprocally bind perception and action. For the sake of simplicity, in the following descriptions we will refer to motor action only.

Note that in this case the person should also show subtle motor deficits.
